# When perception reflects reality: Non‐native grass invasion alters small mammal risk landscapes and survival

**DOI:** 10.1002/ece3.2785

**Published:** 2017-02-15

**Authors:** Joseph P. Ceradini, Anna D. Chalfoun

**Affiliations:** ^1^ Wyoming Cooperative Fish and Wildlife Research UnitDepartment of Zoology and PhysiologyUniversity of WyomingLaramieWYUSA; ^2^ U.S. Geological Survey Wyoming Cooperative Fish and Wildlife Research UnitDepartment of Zoology and PhysiologyUniversity of WyomingLaramieWYUSA

**Keywords:** habitat homogenization, habitat selection, human-induced habitat change, invasion biology, invasive species, optimal foraging, predation risk

## Abstract

Modification of habitat structure due to invasive plants can alter the risk landscape for wildlife by, for example, changing the quality or availability of refuge habitat. Whether perceived risk corresponds with actual fitness outcomes, however, remains an important open question. We simultaneously measured how habitat changes due to a common invasive grass (cheatgrass, *Bromus tectorum*) affected the perceived risk, habitat selection, and apparent survival of a small mammal, enabling us to assess how well perceived risk influenced important behaviors and reflected actual risk. We measured perceived risk by nocturnal rodents using a giving‐up density foraging experiment with paired shrub (safe) and open (risky) foraging trays in cheatgrass and native habitats. We also evaluated microhabitat selection across a cheatgrass gradient as an additional assay of perceived risk and behavioral responses for deer mice (*Peromyscus maniculatus*) at two spatial scales of habitat availability. Finally, we used mark‐recapture analysis to quantify deer mouse apparent survival across a cheatgrass gradient while accounting for detection probability and other habitat features. In the foraging experiment, shrubs were more important as protective cover in cheatgrass‐dominated habitats, suggesting that cheatgrass increased perceived predation risk. Additionally, deer mice avoided cheatgrass and selected shrubs, and marginally avoided native grass, at two spatial scales. Deer mouse apparent survival varied with a cheatgrass–shrub interaction, corresponding with our foraging experiment results, and providing a rare example of a native plant mediating the effects of an invasive plant on wildlife. By synthesizing the results of three individual lines of evidence (foraging behavior, habitat selection, and apparent survival), we provide a rare example of linkage between behavioral responses of animals indicative of perceived predation risk and actual fitness outcomes. Moreover, our results suggest that exotic grass invasions can influence wildlife populations by altering risk landscapes and survival.

## 1 Introduction

Animals must balance tradeoffs between risk and reward to maximize fitness (Lima & Dill, 1990). The energy gain from foraging, for example, is offset by the costs of predation risk, missed mating opportunities, and metabolic demands (Brown & Kotler, 2004; Lima & Dill, 1990). Individuals attempt to maximize benefits by modifying behaviors such as foraging patterns and habitat selection (Brown & Kotler, 2004). Simultaneously, anthropogenic activities have extensively modified habitats across the globe (Ellis, Goldewijk, Siebert, Lightman, & Ramankutty, 2010). Understanding how such habitat changes influence risk landscapes for native species, and whether altered risk has actual fitness consequences is an important challenge.

Invasive plants can dramatically alter habitats by transforming native plant community composition and structure (Vila et al., 2011). Such habitat modification can indirectly alter the costs of particular behaviors for native species by, for example, altering perceived and/or actual predation risk (Bishop & Byers, 2015; Dutra, Barnett, Reinhardt, Marquis, & Orrock, 2011; Holzer & Lawler, 2015; Pearson, 2009; Schmidt & Whelan, 1999). Changes in predation risk can in turn alter fitness outcomes such as nest (Schmidt & Whelan, 1999) and foraging (Ortega, Greenwood, Callaway, & Pearson, 2014; Pearson, 2009) success, which ultimately affects population persistence. Changes in indicators of perceived risk, however, do not necessarily reflect actual predation risk or fitness outcomes (e.g., Shields, Jenkins, Zollner, & Saunders, 2014). Understanding the extent to which habitat modifications due to invasive plants alters both perceived and actual risk requires simultaneous study of behavioral and fitness responses, which is extremely rare (e.g., Pearson, 2009; Sih, Ferrari, & Harris, 2011).

We used a model invasive plant‐native animal system to assess whether habitat changes by an invasive grass altered perceived predation risk and fitness (apparent survival) for small mammals. Small mammals play important functional roles in many ecosystems (Fleming et al., 2014), so the consequences of invasive plants for small mammals will likely affect multiple trophic levels (Dangremond, Pardini, & Knight, 2010; Pearson & Callaway, 2008). To measure perceived risk, we quantified changes in small mammal foraging behavior and habitat selection in response to exotic grass invasion. Foraging experiments using depletable food patches effectively titrate perceived predation risk because animals should stop foraging when the costs of predation risk outweigh the benefits of foraging, assuming other costs are held constant (Brown, 1988; Brown & Kotler, 2004). The predation costs of foraging can then be quantified as a function of different habitat types, such as native versus invasive (Brown, 1988). Habitat selection can also reflect changes in perceived risk, as animals may attempt to mitigate risk by selecting habitat features that reduce exposure to predators (Brown & Kotler, 2004; Lagos, Contreras, Meserve, Gutierrez, & Jaksic, 1995).

Invasive grasses, in particular, are distributed globally, can transform native habitats (D'Antonio & Vitousek, 1992; Pyšek et al., 2012), affect a broad range of taxa (e.g., birds: Jones & Bock, 2005; small mammals: Litt & Steidl, 2011; ungulates: Kohl, Hebblewhite, Cleveland, & Callaway, 2012; arthropods: Litt, Cord, Fulbright, & Schuster, 2014; reptiles: Gray & Steidl, 2015; amphibians: Holzer & Lawler, 2015), and will likely become increasingly dominant as they are favored over native species due to positive feedbacks (Mack & Antonio, 1998) and, likely, climate change (Blumenthal, Kray, Ortmans, Ziska, & Pendall, 2016; Smith et al., 2000). Cheatgrass (*Bromus tectorum*), one of the most widespread invasive plants in North America (Bradley & Mustard, 2005), can drastically alter wildlife habitat by replacing native plant communities with dense grass cover, thereby simplifying plant community composition and structure (Knapp, 1996). Habitat changes by cheatgrass can decrease small mammal abundance and diversity (Freeman et al., 2014; Ostoja & Schupp, 2009); however, there is a limited understanding of how cheatgrass alters risk landscapes (e.g., Rieder, Newbold, & Ostoja, 2010). Finally, we are aware of only one study that assessed the fitness consequences of cheatgrass invasion for small mammals (Steenhof, Yensen, Kochert, & Kenneth, 2006).

We assessed small mammal foraging behavior and habitat selection in relation to cheatgrass and other habitat elements frequently documented to alter predation risk. There is strong experimental evidence that small mammals alter foraging behavior and habitat selection in response to varying levels of risk (Kotler, Brown, Mukherjee, Berger‐Tal, & Bouskila, 2010; Lagos et al., 1995; Orrock & Danielson, 2004). Small mammals frequently select microhabitats such as shrubs over riskier open habitats, suggesting that shrubs decrease risk (Lagos et al., 1995; Longland & Price, 1991; however, see Parmenter & Macmahon, 1983). Small mammals also often forage less when the moon is brighter (Prugh & Golden, 2014; however, see Upham & Hafner, 2013), suggesting that prey may be more visible, and thus more susceptible to predation, under brighter moons. We used these relationships to develop testable predictions for how cheatgrass influenced perceived risk for small mammals.

Invasive grasses can increase predation risk for small mammals by impeding their movement (Rieder et al., 2010) and increasing the volume of noise created by rodents moving through vegetation (Bachen, 2014), which may hinder predator avoidance. We thus predicted that shrubs should be more important as protective cover, and bright moons should be riskier, for small mammals as cheatgrass cover increased. Conversely, if dense invasive grass decreases predation risk by providing protective cover (Johnson & De León, 2015), we expected the effects of shrubs and moonlight to be dampened or unchanged within invaded habitats. Alternatively, changes in food quality or quantity due to non‐native grass invasion may alter small mammal foraging behavior and habitat selection. Cheatgrass may provide food for small mammals directly through seeds (Richardson, West, & Gitzen, 2013) and indirectly by increasing arthropod abundance (Litt et al., 2014; Ostoja, Schupp, & Sivy, 2009). Cheatgrass seeds are lower in nutritional quality, however, and due to persistent awns, likely require greater handling time than many native seeds (Kelrick, Macmahon, Parmenter, & Sisson, 1986; Lucero, Allen, & McMillan, 2015). Finally, cheatgrass and native grass may provide similar habitat structure for small mammals in certain ecological contexts (Martin & Murray, 2011). We thus also assessed the effect of native grass, and cheatgrass while controlling for native grass, on foraging behavior and habitat selection.

We quantified the costs of foraging for nocturnal rodents in relation to cheatgrass, native grass, shrubs, and moonlight using a giving‐up density experiment (GUD), which is a common approach for assessing predation risk (Brown, 1988; Brown & Kotler, 2004). We used powder tracking to quantify microhabitat selection at two spatial scales for a common small mammal in North America, the deer mouse (*Peromyscus maniculatus*), in relation to cheatgrass, native grass, shrubs, and moonlight. Finally, we assessed deer mouse apparent survival using mark‐recapture analysis. To our knowledge, this is the first study to experimentally assess changes in perceived risk and simultaneously measure behavioral and fitness responses of small mammals as a result of habitat modification by invasive plants.

## 2 Materials and Methods

### 2.1 Study area

Field work was conducted in Thunder Basin National Grassland (TBNG), WY, which is a semi‐arid grassland managed by the US Forest Service (average annual precipitation = 31.75 cm, range = 14.22–49.53; elevation range = 1097–1585 m) (Haufler, Mehl, Ganguli, & Yeats, 2008; USFS 2011). Vegetation was primarily mixed‐grass prairie with a substantial shrub component comprised of big sagebrush (*Artemisia tridentata*) or greasewood (*Sarcobatus vermiculatus*) (Haufler et al., 2008). The dominant native herbaceous plants were western wheatgrass (*Elymus smithii*), blue grama (*Bouteloua gracilis*), and needle‐and‐thread (*Hesperostipa comata*). Cheatgrass was the dominant invasive plant followed by Japanese brome (*Bromus japonicus*) (Haufler et al., 2008), which was much less common.

We selected 16 sites that spanned a gradient of cheatgrass and shrub cover while minimizing variation in other site characteristics, such as topography (Table 2). Median minimum distance between sites was 872 m (range = 207–8794). Vegetation cover was estimated with line point intercept surveys (Bonham, 2013) along eight 20 m transects distributed in a random, spatially balanced pattern at each site. Transects were separated by at least 20 m within a site. Riparian areas may act as a small mammal population source for adjacent upland habitats (Hamilton, Roeder, Hatch, Eggett, & Tingey, 2015). No site contained riparian habitat; however, some sites were adjacent to riparian zones (≥5 m). To account for a potential riparian effect, the proportion of riparian habitat within a 200 m buffer of each site was calculated in ArcMap (Environmental Systems Resource Institute 2012) (Table 2, Appendix S1). We counted piles of cow dung on eight 20 × 1.5 m belt transects on each site to assess relative cattle use (Table 2), which may influence small mammals by altering vegetation structure and composition (Bueno, Ruckstuhl, Arrigo, Aivaz, & Neuhaus, 2012). Data on daily moon illumination were obtained from the US Naval Observatory database (2014). We obtained daily temperature and precipitation data from the Rochelle Hills weather station in TBNG (mean distance from sites: 21 km; range: 5–31). Cloud cover data were not available for our sites (see Appendix S1 for additional study area and site selection details).

### 2.2 Foraging costs experiment

We assayed perceived predation risk in 2014 by measuring the foraging behavior of nocturnal rodents using the giving‐up density (GUD) in paired foraging trays (Brown, 1988). The foraging experiment was implemented at a subset of sites that had similar shrub cover but varied in cheatgrass cover: four sites with high and four sites with low cheatgrass cover (Table 2). The experiment ran on each site for three consecutive nights during the moon's lower quarter (0–25% illumination) and three consecutive nights during roughly the upper quarter (68–100% illumination). Foraging trays were simultaneously set on two high and two low cheatgrass sites to minimize the effects of possible confounding factors such as weather.

Each site received seven pairs of foraging trays per night (8 sites × 6 nights × 7 tray pairs = 336 tray pair nights); pairs were separated by a minimum of 40 m to reduce the likelihood of an individual small mammal visiting more than one pair. Each pair contained one tray placed under a shrub (safe tray) and one tray 1.5 m away in the open (risky tray). To determine the location of each pair of trays, we generated a grid of 40 m buffered points for each site (Environmental Systems Resource Institute 2012) and selected the closest suitable juxtaposition of shrub and open microhabitats to the point. At high cheatgrass cover sites, non‐shrub trays were placed in dense cheatgrass, whereas at low cheatgrass cover sites non‐shrub trays were placed in the dominant habitat, which was often a mix of sparse native grass, cheatgrass, and bare ground (Figure S1). Foraging trays were 35.9 × 20 × 12.4 cm clear plastic storage containers (Sterilite Inc.) with latching translucent lids affixed (Mattos & Orrock, 2010). A 6 cm diameter hole, 3.5 cm from the tray bottom, was drilled on two opposing sides of the container to facilitate rodent access. Each tray was filled with 5.0 g of sterilized sunflower seeds mixed into 1 L of play sand (Pavestone Inc.; Jacob & Brown, 2000).

Trays were set with sand–seed mixtures at approximately sunset and checked the following morning starting 1 hr before sunrise. There were no signs of bird foraging at any tray. Nightly replacement of mixtures was impractical given the number of tray nights (672). Sand–seed mixtures were replaced, however, if the tray was visited, disturbed, damp or wet, or the sand was compacted due to settling. Otherwise, mixtures were remixed and used again the following night. Sand from visited trays was sifted and remaining seeds were dried in an oven for 24 hr at 60°C and weighed.

As a rodent depletes the seeds in a tray, the costs of foraging, such as predation risk, eventually outweigh the benefits and the rodent should stop foraging (Brown, 1988). This threshold is measured as the giving‐up density (GUD). The paired tray design is critical because potential confounding factors, such as background food availability, and predator density and assemblage, are assumed to be similar for each pair of trays. Thus, assuming that potential confounding factors are comparable within a pair, differences in GUDs between paired risky and safe trays should represent a quantitative measure of perceived predation risk (Brown, 1988; Brown & Kotler, 2004).

### 2.3 Habitat selection

Deer mouse habitat selection was assessed in 2013 and 2014 using powder tracking following Stapp (1997) (Table 2; see Appendix S2 for additional details on this method). On the final morning of a primary trapping period, a subset of deer mice (excluding lactating or pregnant females) were removed from the site and placed in a quiet, cool location during the day. Approximately 1–2 hr before sunset, mice were coated with fluorescent powder (Radiant Color, Inc.) and released at the location of capture. To target nocturnal rather than initial escape behavior, the track was first followed at approximately sunset in order to mark the last daytime location, which was often a burrow. Starting from the last daytime location, the track was followed beginning 2–4 hr after sunset using an ultraviolet LED flashlight (https://ledwholesalers.com) and flagged every meter.

Every flag or used point on the track was paired with an unused point. To quantify available microhabitat within an ecologically meaningful distance of the track, available points were randomly located between 1 and 5 m from used points and alternated between the left and right side of the track (Stapp, 1997). For each track, used and available plant cover was estimated by treating the “tracks” as transects and conducting a line point intercept survey at each point, following the same protocol as Site‐level vegetation surveys (Table 1; Bonham, 2013). Site‐level cover estimates represented available macrohabitat.

**Table 1:** Predictor names and descriptions for all analyses

Predictor	Description
*Habitat variables*	
Cheat	Cheatgrass cover (%)
Cheat.cat	Categorical cheatgrass (high = 1, low = 0)
Shrub	Shrub cover (%)
Natv.g	Native grass cover (%)
Riparian	Adjacent riparian habitat (%)
Cattle	Cattle use index (count)
*Abiotic variables*	
Moon	Moon illumination (%)
Precip	Precipitation (mm)
Low.temp	Low temperature (F)
*Control variables*	
Abundance	Estimated deer mouse abundance
Null	Constant (intercept‐only)
Site	Trapping grid
Year	2014 = 1, 2013 = 0
Effort	Corrected number of trap nights^a^

a Corrected number of trap nights was calculated following Beauvais and Buskirk (1999).

### 2.4 Apparent survival

To estimate survival, we used a robust trapping design (Williams, Nichols, & Conroy, 2002) with four primary trapping periods (early to mid‐summer and mid‐ to late‐summer in 2013 and 2014) each consisting of four secondary periods (trap nights). Within a year, primary periods were separated by approximately 1 month (range: 30–39 days), which represented the survival interval. There were four blocks that each contained four sites spanning a gradient of cheatgrass cover (Table 2). To better isolate the effect of cheatgrass, we simultaneously trapped all sites within a block to control for temporal autocorrelation due to factors such as weather, and variability in the survival interval (the survival interval was always the same for sites within a block; Table 2). This trapping design also allowed for estimation of deer mouse abundance, while accounting for detection probability, for each site and primary period combination.

**Table 2:** Location of each field component and summarized site covariate values

Site	Apparent survival	Foraging behavior	Habitat selection	Cheat (%)	Native grass (%)	Shrub (%)	Cattle (count)	Riparian (%)
B1‐1	Y	N	0	1 (1)	54 (5)	0 (0)	1 (1)	0
B1‐2	Y	N	3	2 (2)	56 (4)	0 (0)	2 (1)	0
B1‐3	Y	N	0	16 (2)	61 (6)	0 (0)	11 (2)	0
B1‐4	Y	N	3	24 (4)	52 (5)	0 (0)	5 (1)	0
B2‐1	Y	Y	3	4 (2)	18 (3)	11 (2)	9 (1)	6
B2‐2	Y	Y	2	7 (2)	36 (5)	9 (1)	7 (1)	0
B2‐3	Y	Y	3	53 (5)	49 (3)	25 (3)	3 (0)	12
B2‐4	Y	Y	4	53 (5)	47 (4)	24 (3)	3 (1)	14
B3‐1	Y	Y	3	0 (0)	55 (3)	16 (3)	0 (0)	4
B3‐2	Y	Y	4	1 (1)	68 (4)	16 (2)	4 (0)	3
B3‐3	Y	Y	3	24 (6)	59 (4)	12 (2)	3 (0)	3
B3‐4	Y	Y	4	71 (3)	43 (4)	21 (4)	8 (2)	0
B4‐1	Y	N	2	1 (1)	57 (5)	0 (0)	2 (0)	0
B4‐2	Y	N	0	7 (2)	96 (1)	4 (2)	5 (2)	13
B4‐3	Y	N	0	20 (6)	52 (8)	0 (0)	9 (2)	5
B4‐4	Y	N	4	26 (6)	34 (5)	3 (1)	5 (1)	10

The 16 sites were divided into four blocks, B1–B4. Y and N under Apparent survival and Foraging behavior indicate if the component was implemented (Y) or not (N) on the site. Habitat selection is the count of individual deer mice that were powder tracked. Apparent survival and habitat selection were assessed in 2013 and 2014, whereas foraging behavior was only assessed in 2014. None of the field components occurred on the same nights. Cheat (cheatgrass), native grass, and shrub are mean percent cover estimates from line point intercept surveys. Cattle is the mean count of cow dung piles. Standard errors are in parentheses. Riparian is the proportion of riparian habitat within a 200 m buffer of each site and does not have a SE. Within a block, the table is sorted by Cheat. All information was pooled across years to facilitate interpretation

Each site had a 135 × 165 m trapping grid consisting of 120 live traps separated by 15 m. The grid configuration of four sites was slightly modified to stay within the primary habitats (trap spacing was unaltered). Each grid had 80 Sherman (H.B. Sherman Traps Inc.) and 40 Havahart traps (Woodstream Corp.). Grids contained two Sherman traps followed by one Havahart trap in a repeating pattern, with one trap per station. Captures were marked with a PIT tag (passive integrated transponder, Biomark Inc.) and released at the location of capture (see Appendix S3 for additional trapping details).

### 2.5 Analysis methods

We used a multi‐model inference approach to assess the relative support for our primary predictions (Burnham & Anderson, 2002). To minimize the effects of multicollinearity, we used a cutoff of two for the variance inflation factor. All interactions represented *a priori* hypotheses and models with interactions included main effects. For the foraging costs and survival analyses, we used Akaike's Information Criterion corrected for small sample sizes (AIC_*c*_) for model selection. AIC_*c*_ weight and ∆AIC_*c*_ were used to compare the relative support for each model (Burnham & Anderson, 2002; Mazerolle, 2015). Model selection for the habitat selection analysis was conducted with the quasi‐likelihood under independence criterion (QIC(I)), which is appropriate for models fit with quasi‐likelihood methods such as conditional logistic regression (Craiu, Duchesne, & Fortin, 2008; Pan, 2001). QIC can be considered a generalization of AIC; thus, we interpret QIC rankings similar to AIC (Craiu et al., 2008; Pan, 2001).

We considered models to be uninformative if a nested model with one less predictor differed in negative log likelihood by ≤1 and was within 0–3.5 ∆AIC_*c*_ (Arnold, 2010; Burnham & Anderson, 2002; Murtaugh, 2014). Models with uninformative predictors are presented in final model sets to facilitate interpretation; however, such models were excluded when calculating AIC_*c*_ weights. All analyses and plotting were done in program R (R Core Team 2015; Wickham, 2009).

#### 2.5.1 Foraging costs experiment

We used the differences in GUD between paired open and shrub trays (open GUD – shrub GUD) as the response variable in a linear‐mixed model (LMM) (Orrock & Fletcher, 2014). Because the GUD experiment was implemented on a subset of sites with primarily high or low cheatgrass cover (Table 2), cheatgrass was modeled as a categorical predictor (*cheat.cat*, Table 1). Nightly variation in paired GUD differences within a moon stratum was not central to our questions; thus, differences for each pair were averaged across the three nights within the moon's lower quarter and the three nights in roughly the upper quarter (*n* = 62 after averaging). Time‐varying covariates, such as temperature, were averaged likewise. Pairs were excluded from analysis if neither tray was visited during a given night (Mattos & Orrock, 2010). Temporal consistency in plots of nightly GUD for each site suggested that rodents readily acclimatized to the novel foraging trays; thus, all nights were included in analysis.

Although all tray pairs within a site were separated by a minimum of 40 m, we expected pairs within a site to be more correlated than pairs between sites. We thus included a random intercept for site in all models (Zuur, Ieno, Walker, Saveliev, & Smith, 2009). There is no consensus on how to calculate degrees of freedom for mixed models (Bolker et al., 2009). We therefore did not report *p* values and assessed predictor significance based on bootstrapped 95% confidence intervals and *t* values (e.g., Doherty, Davis, & van Etten, 2015). Goodness of fit was evaluated on the residuals and random effects (Zuur et al., 2009), and model performance was measured by the marginal ($R_{m}^{2}$) and conditional ($R_{c}^{2}$) coefficients of determination (Barton, 2015; Nakagawa & Schielzeth, 2013).

If cheatgrass altered predation risk, we predicted that the effect of shrub cover and/or moonlight on rodent foraging behavior should depend on level of cheatgrass cover. Thus, the primary tests of our altered predation risk predictions were a cheatgrass–shrub cover interaction and a cheatgrass–moonlight interaction. We also evaluated whether nocturnal rodent abundance influenced the paired differences. Abundance, however, is more likely to affect absolute GUD than paired GUD differences (Orrock & Fletcher, 2014).

#### 2.5.2 Habitat selection

We used conditional (or matched case‐control) logistic regression to assess habitat selection (Craiu et al., 2008; Hosmer, Lemeshow, & Sturdivant, 2013; Therneau, 2015). For our study design, this approach was more ecologically meaningful and statistically powerful than standard unconditional logistic regression. Conditional logistic regression enabled us to pair used and available habitat by individual deer mouse, rather than comparing all used habitat to all available habitat. We used site as a cluster to account for higher expected correlation between individuals within than among sites (*n* = 35 adults and 3 sub‐adults, strata = individual, cluster = site). Consistent with the foraging behavior analysis, the interaction between cheatgrass and shrub cover, and cheatgrass and moonlight, were the primary tests of our altered predation risk predictions for habitat selection. We only retained sex and male reproductive status (only non‐reproductively active females were included) predictors if they were significant.

Habitat selection is often a hierarchical process that occurs at nested spatial scales (Chalfoun & Martin, 2007; Johnson, 1980). For micro‐selection, used microhabitat was paired with available habitat within a 5 m buffer of each track. For macro‐selection, used microhabitat was paired with Site‐level cover estimates (see Appendix S2 for additional modeling details).

#### 2.5.3 Apparent survival

We used the Huggins robust design, with a logit link function, to estimate capture (*p*) and recapture (*c*) probability, apparent survival (*Φ*), and abundance ($\hat{N}$) for deer mice (effective *n* = 2409; Williams et al., 2002). Temporary emigration parameters (γ^*ʹ*^ and γ″) were fixed to zero because there were only two primary periods per year and survival was near zero between years. We thus estimated apparent survival, which may be an underestimate of true survival, since emigration and death are confounded when γ^*′*^ and γ″ are inestimable (Williams et al., 2002).

We used a two‐stage approach (Doherty, White, & Burnham, 2012) to model detection and apparent survival probability. In stage one, apparent survival was held constant as a global model while different detection models competed. In stage two, different apparent survival models competed while detection was held as the top model from stage one. If there was model selection uncertainty in stage one, competitive models moved forward to compete again in stage two. All inference was based on the stage two model set (see Appendix S3 for additional details).

## 3 Results

### 3.1 Predation costs of foraging

**Figure 1:**
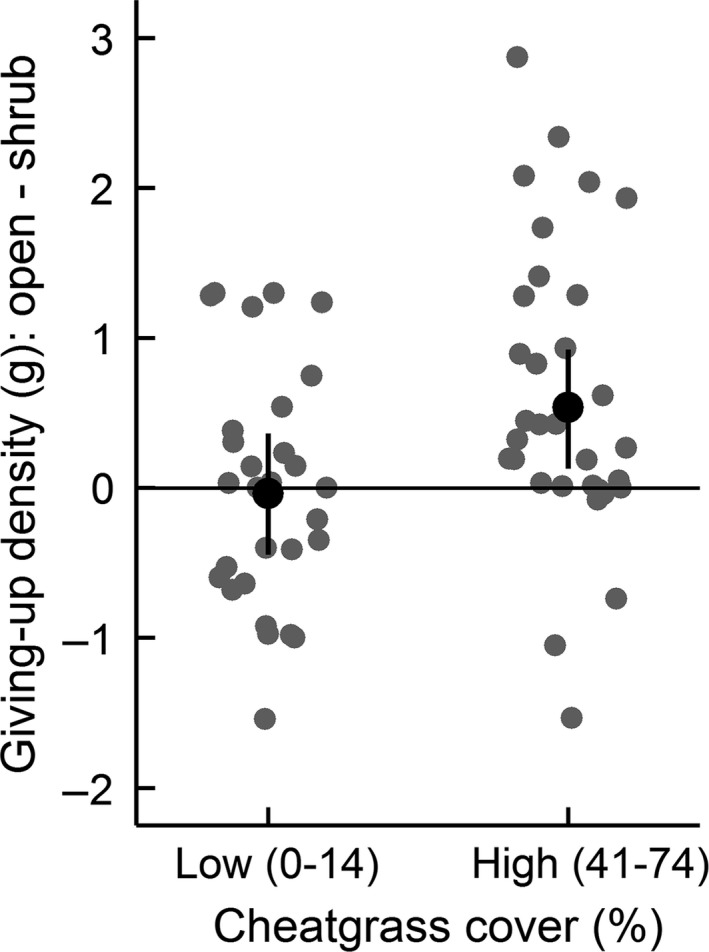
Paired giving‐up density differences (open tray GUD – shrub tray GUD) as a function of categorical cheatgrass and continuous native grass predictors. Black points are the predicted mean difference with bootstrapped 95% confidence intervals from the top GUD model. Native grass was held at its mean for predictions. Gray points are observed paired differences and are jittered. GUD was higher in the open than shrub tray for points above the horizontal line, and vice‐versa for points below the horizontal line

The paired GUD difference for shrub and open trays was contingent on the level of cheatgrass cover, with more seed removed from shrub trays where cheatgrass was dominant. There was a significant interaction between cheatgrass and shrub cover in the top two GUD models (Table 3). The direction of the interactions supported the prediction that cheatgrass increased perceived predation risk. Because the response variable was (open tray GUD – shrub tray GUD), the intercept in the top model represents the shrub effect when cheatgrass and native grass were low, which was not significant (β = −0.83, 95% CI = −1.88 to 0.32, *t *= −1.56; Figure 1). The categorical cheatgrass predictor is the difference between the shrub effect when cheatgrass was high versus low, and suggests the shrub effect was significantly stronger where cheatgrass was high (β = 0.57, 95% CI = 0.03 to 1.10, *t *=* *2.04; Figure 1). The native grass effect was inconsistent (β = 0.16, 95% CI = −0.03 to 0.34, *t *=* *1.65), however, it improved model performance based on $R_{m}^{2}$ (Table 3). Moreover, the second ranked model only contained the categorical cheatgrass term (β = 0.68, 95% CI = 0.08 to 1.26, *t *=* *2.20) and suggests the same shrub–cheatgrass interaction in support of the prediction that perceived risk increased with cheatgrass. The native grass terms in the third and fourth ranked models, and the cheatgrass–moon interaction, were not significant.

**Table 3:** Foraging behavior experiment model set from a linear‐mixed model with (open tray GUD – shrub tray GUD) as the response variable

Model	*K*	∆AIC_c_ a	*w*	$R_{m}^{2}$	$R_{c}^{2}$	LL
Cheat.cat + natv.g	5	0.00	0.36	0.18	0.19	−77.55
Cheat.cat	4	0.95	0.22	0.13	0.18	−79.20
Natv.g^2^	5	2.36	0.11	0.14	0.17	−78.73
Natv.g	4	2.40	0.11	0.09	0.16	−79.93
Cheat.cat + low.temp	5	3.29	NA	0.13	0.18	−79.19
Null	3	3.49	0.06	0.00	0.17	−81.62
Cheat.cat × precip	6	4.11	NA	0.15	0.21	−78.37
Shrub	4	4.23	0.04	0.05	0.16	−80.85
Cheat.cat × moon	6	5.47	NA	0.13	0.19	−79.05
Moon	4	5.50	0.02	0.00	0.17	−81.48
Precip	4	5.65	0.02	0.00	0.16	−81.55
Abundance	4	5.66	0.02	0.00	0.16	−81.56
Low.temp	4	5.78	0.02	0.00	0.17	−81.62
Shrub × moon	6	6.21	0.02	0.09	0.23	−79.42
Natv.g × moon	6	6.63	NA	0.10	0.17	−79.63
Global	14	15.90	0.00	0.30	0.30	−72.56

AIC_*c*_ = Akaike's Information Criterion corrected for small sample sizes. Models with NA under *w* meet the uninformative predictor criteria. All models contained a random intercept term for site. *K *= number of parameters, ∆AIC_*c*_ = AIC_*ci*_ − minimum AIC_*c*_, *w *= AIC_*c*_ model weight, $R_{m}^{2}$ and $R_{c}^{2}$ = marginal and conditional *R* ^2^, respectively, and LL = log‐likelihood.

a Minimum AIC_*c*_ = 166.16.

### 3.2 Habitat selection

**Figure 2:**
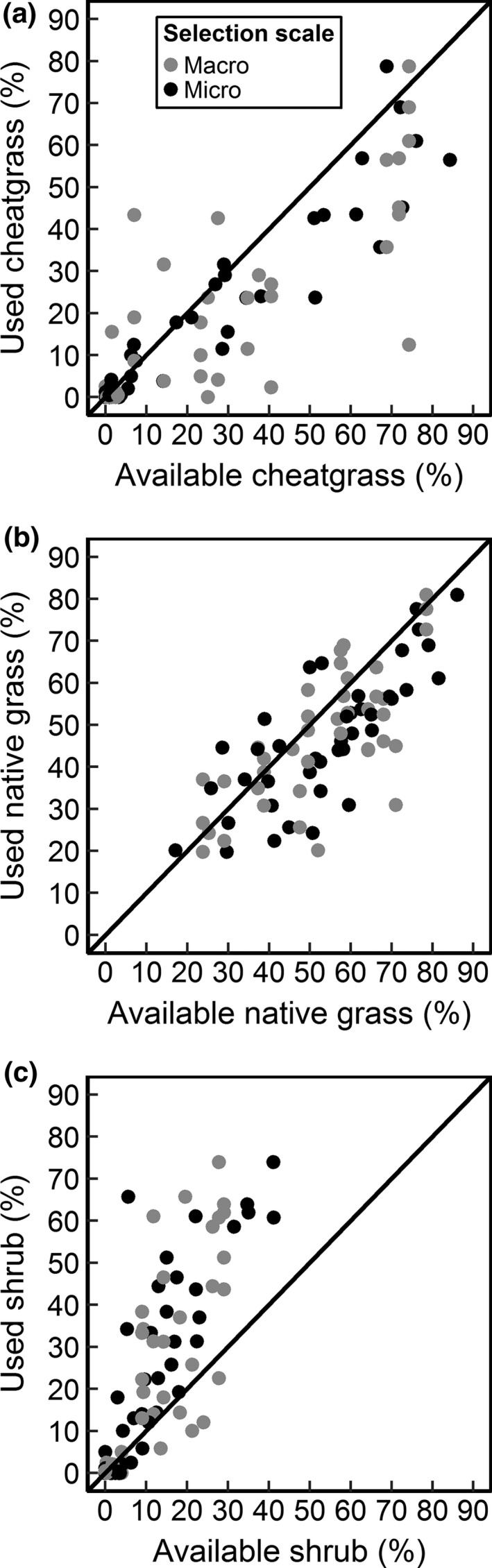
Observed ratios of used (based on powder tracking) to available cheatgrass (a) native grass (b), and shrub (c) cover for deer mice. Used microhabitat was compared to available microhabitat (track scale) and used microhabitat was compared to available macrohabitat (site scale). Points are individual deer mice and each mouse is represented once for each spatial scale in a, b, and c. Lines are 1:1 lines thus individuals below the line used the cover less than it was available (avoidance) while individuals above the line selected the cover type

Deer mice avoided dense cheatgrass and selected shrubs at both scales of habitat assessment (Figure 2; Tables 4 and 5). Deer mice also avoided areas with high cover of native grass at both spatial scales; however, the cheatgrass and shrub effects were much stronger (Figure 2; Table 5). Nonetheless, the addition of native grass to the additive cheatgrass–shrub model substantially improved model performance (Table 4). The cheatgrass–shrub interaction, representing an altered perceived risk prediction, was not significant at either spatial scale (micro–micro: β = 0.37, 95% CI = −0.11 to 0.85, *p *=* *0.13; micro–macro: β = 0.03, 95% CI = −0.12 to 0.18, *p *=* *0.69), and neither interaction improved model performance relative to a simpler additive cheatgrass–shrub model (Table 4). The cheatgrass–moon interaction was significant at the micro (β = 0.67, 95% CI = 0.06–1.31, *p *=* *0.03) but not macro (β = 1.18, 95% CI = −0.47 to 2.83, *p *=* *0.16) scale. The micro‐scale cheatgrass–moon interaction suggested that cheatgrass was significantly avoided in both the lower and upper quarter of moon illumination; however, avoidance was significantly more negative in the lower quarter. This result partially supports the decreased perceived risk prediction; however, this model had little QIC(I) support and low explanatory power (Table 4). The sex and male reproductive status predictors were not significant, and therefore excluded from the final model set (Table 4).

**Table 4:** Conditional logistic regression model sets for deer mouse habitat selection at two spatial scales

Model	*K*	∆QIC (I)	QIC (I)	C	Quasi‐LL
*Micro used vs. micro available*					
Cheat + natv.g + shrub	3	0.00	25.50	0.87	−9.75
Natv.g + shrub	2	1.75	27.25	0.84	−11.62
Cheat + shrub	2	3.65	29.14	0.76	−12.57
Cheat × shrub	3	4.78	30.27	0.76	−12.14
Shrub	1	5.24	30.73	0.74	−14.37
Global	8	6.74	32.23	0.89	−8.12
Cheat + natv.g	2	6.83	32.32	0.76	−14.16
Natv.g	1	14.58	40.07	0.76	−19.04
Cheat × moon	2	16.26	41.75	0.64	−18.88
Cheat	1	17.22	42.72	0.64	−20.36
Cheat × precip	2	18.71	44.21	0.64	−20.10
Cheat^2^	2	19.18	44.68	0.64	−20.34
*Micro used vs. macro available*					
Cheat + natv.g + shrub	3	0.00	28.07	0.87	−11.03
Cheat + shrub	2	2.97	31.03	0.82	−13.52
Cheat × shrub	3	4.89	32.96	0.82	−13.48
Global	8	5.12	33.19	0.87	−8.59
Natv.g + shrub	2	5.79	33.86	0.84	−14.93
Shrub	1	9.92	37.99	0.68	−17.99
Cheat + natv.g	2	12.92	40.99	0.84	−18.49
Natv.g	1	17.05	45.12	0.70	−21.56
Cheat × moon	2	18.82	46.89	0.72	−21.44
Cheat	1	19.18	47.25	0.72	−22.63
Cheat^2^	2	20.05	48.12	0.72	−22.06
Cheat × precip	2	20.88	48.95	0.72	−22.47

For all models, strata = individual and cluster = site. QIC(I) = quasi‐likelihood under independence criterion, *K* = number of parameters, ∆QIC(I) = QIC(I)_*i*_ − minimum QIC(I)*,* C = concordance, and Quasi‐LL = quasi‐log‐likelihood.

**Table 5:** Back‐transformed coefficient estimates from top deer mouse habitat selection models. Models were fit with conditional logistic regression

Predictor	OR	LCL	UCL	*p*
*Micro used vs. micro available* a				
Cheatgrass	0.08	0.02	0.31	0.0002
Native grass	0.30	0.08	1.05	0.0592
Shrub	1.85	1.22	2.81	0.0037
*Micro used vs. macro available* b				
Cheatgrass	0.34	0.16	0.76	0.0080
Native grass	0.41	0.18	0.96	0.0407
Shrub	2.27	1.57	3.27	<0.0000

The cheatgrass and native grass estimates are the effect of a 10% increase in cover, whereas the shrub estimates are the effect of a 5% increase in cover. OR = odds ratio or exp(β), LCL and UCL = lower and upper 95% confidence limit of OR, and *p *= *p*‐value.

a The cheatgrass, native grass, and shrub effects were also significant in the second and third ranked micro–micro habitat selection models.

b The cheatgrass, native grass, and shrub effects were also significant in the second and fifth ranked micro–macro habitat selection models.

### 3.3 Apparent survival

**Figure 3:**
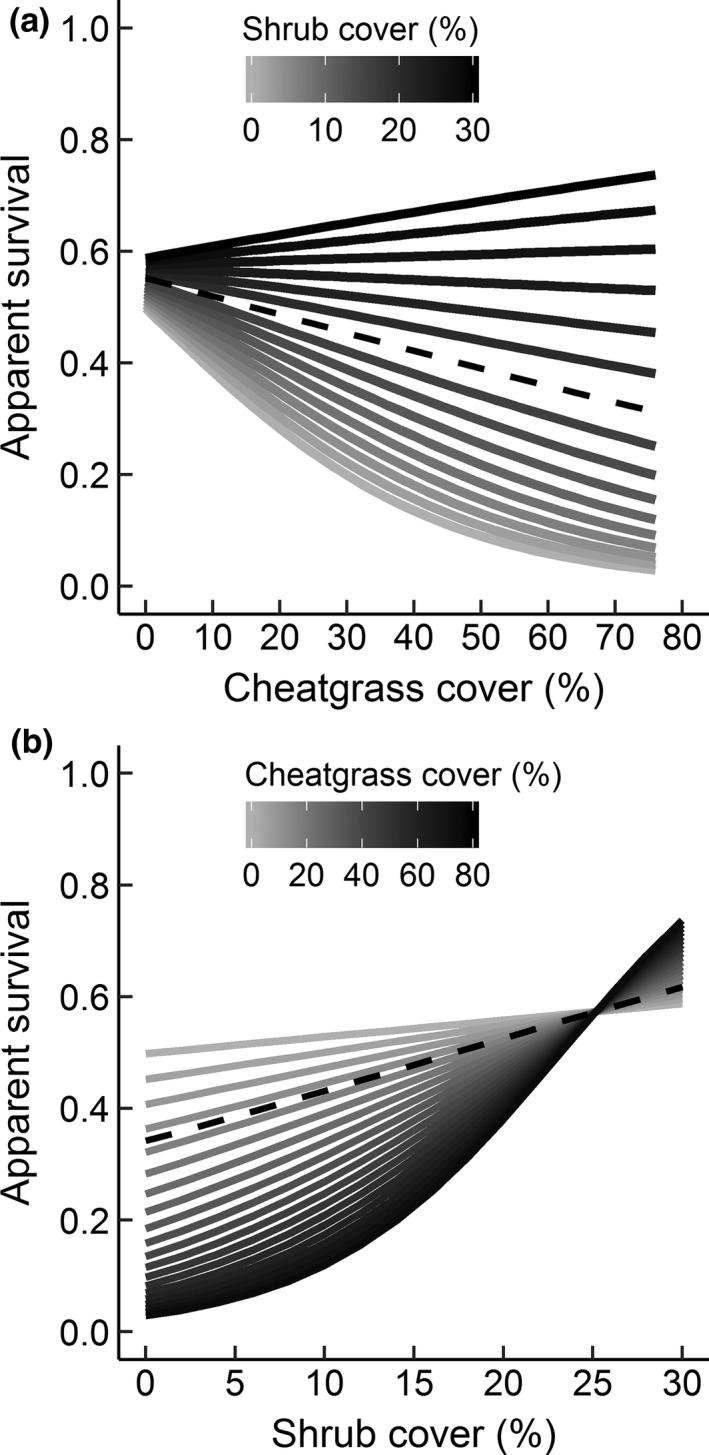
Predicted apparent survival for deer mice across a gradient of cheatgrass and shrub cover (a, b) based on the top model, *cheat *×* shrub + year*. The cheatgrass effect in (a) was significantly negative when shrub cover was less than 18% (black dashed line). The shrub effect in (b) was significantly positive when cheatgrass cover was greater than 14% (black dashed line). Predictions shown are for 2014

The deer mouse apparent survival results paralleled the GUD experiment findings. Cheatgrass had a negative or neutral effect, and shrub cover had a positive or neutral effect, on monthly apparent survival of deer mice. The top apparent survival model had moderate AIC_*c*_ support (Table 6; Table S1; evidence ratio = 2.35) and contained a positive interaction between cheatgrass and shrub cover (β = 0.09, 95% CI = 0.004 to 0.18, *z *=* *2.05; Figure 3). Cheatgrass had a negative effect on apparent survival when shrub cover was <18% (Figure 3). When shrub cover was 0%, for example, the odds of apparent survival decreased by 37% (95% CI = 6 to 58) for every 10% increase in cheatgrass cover. The effect of cheatgrass on apparent survival was equivocal when shrub cover was >18%.

**Table 6:** Subset of stage 2 deer mouse apparent survival (Φ) model set from the Huggins robust design analysis

Model	*K*	∆AIC_c_ a	*w*	Deviance
Cheat × shrub + year	27	0.00	0.47	6697.22
Cheat^2^ + year	26	1.70	0.20	6700.97
Cheat^2^ + shrub + year	27	2.11	0.16	6699.32
Cheat + shrub + year	26	2.23	0.15	6701.49

AIC_*c*_ = Akaike's Information Criterion corrected for small sample sizes. For all models, probability of capture (*p*) = year + age + sex and recapture (*c*) = year + site + age + sex. *K *= number of parameters, ∆AIC_*c*_ = AIC_*ci*_ –minimum AIC_*c*_, and *w *= AIC_*c*_ model weight

a Minimum AIC_*c*_ = 6751.85.

Apparent survival of deer mice increased significantly with shrub cover only when cheatgrass cover was >14%, otherwise the shrub effect was inconsistent (Figure 3). When cheatgrass cover was 40%, for example, the odds of apparent survival increased by a factor of 1.53 (95% CI = 1.26 to 1.87) for every 5% increase in shrub cover. Apparent survival did not vary with year (exp(β) = 2.15, 95% CI = 0.69 to 6.75, *z *=* *1.33). The second ranked model was competitive and contained a positive quadratic effect of cheatgrass on apparent survival (β = 0.10, 95% CI = 0.05 to 0.15, *z *=* *3.84; Table 6).

## 4 Discussion

Habitat structure can be a significant determinant of predation risk for animals (Chalfoun & Martin, 2009; Dutra et al., 2011). Non‐native plant invasion can alter habitats and therefore, potentially, risk landscapes and fitness outcomes for native species (Dutra et al., 2011; Pearson, 2009; Schmidt & Whelan, 1999). Few studies, however, have attempted to link changes in perceived risk due to exotic plant invasion to actual risk, which requires simultaneously quantifying changes in behavioral responses and fitness.

We used a model invasive plant–animal system to assess whether habitat changes by exotic plants can alter risk and apparent survival. In a giving‐up density (GUD) foraging experiment, shrubs were more important as protective cover for nocturnal rodents in cheatgrass‐dominated habitats, suggesting that cheatgrass increased perceived risk. Deer mice also avoided cheatgrass and selected shrubs, and marginally avoided native grass, at both the micro‐ and macro‐scales of habitat availability. Finally, the positive effect of shrubs on apparent survival was stronger as cheatgrass cover increased, whereas the negative effect of cheatgrass on apparent survival increased as shrub cover decreased. All three metrics—foraging behavior, habitat selection, and apparent survival—therefore suggested a negative cheatgrass and positive shrub effect, providing strong evidence that cheatgrass increased predation risk and resulted in negative fitness consequences for small mammals.

### 4.1 Effects of cheatgrass on perceived risk

Invasive plants can simultaneously alter food resources (plant composition) and protective cover (plant structure), making it difficult to identify the mechanisms underlying changes in animal populations and communities (Dutra et al., 2011). Our GUD foraging experiment suggested that perceived risk increased with changes in habitat structure due to cheatgrass, regardless of local food availability, and conspecific or predator densities. The dense cover created by cheatgrass may impede movement of small mammals (Rieder et al., 2010), which may render them more vulnerable to predation. Additionally, cheatgrass increases litter depth (Belnap & Phillips, 2001), which also may hinder small mammal movement (Reed, Kaufman, & Kaufman, 2005). The costs of foraging away from substantial cover, such as shrubs, may be greater in cheatgrass‐dominated habitats where it may be harder for small mammals to run to escape cover.

Invasive grasses may also function as protective cover in particular ecological contexts. Johnson and De León (2015) found lower perceived predation risk (based on GUD) by small mammals in sand dunes with dense cover of the invasive European beachgrass (*Ammophila arenaria*) than in sand dunes with sparse native plants. Although European beachgrass may also impede movement (Rieder et al., 2010), the net effect of the invasion may be to reduce predation risk relative to the sparsely vegetated native dunes. Fitness metrics were not concurrently estimated, however, so the lower perceived risk in the invaded dunes may not have reflected actual risk (Johnson & De León, 2015). The value of refuge habitat is also likely contingent on the availability of other forms of cover (Bishop & Byers, 2015). Native shrubs provided substantial cover in our system, potentially lowering the relative value of cheatgrass as escape cover, and resulting in a net increase in predation risk in cheatgrass habitats (Bishop & Byers, 2015).

The costs of foraging should be lower for individuals in poorer condition because the marginal value of food is higher (Brown & Kotler, 2004). We do not know the condition of individuals that visited foraging trays, which could have biased our GUD results (Bedoya‐Perez, Carthey, Mella, McArthur, & Banks, 2013); however, the bias would likely be conservative or negligible. Cheatgrass seeds are likely a lower quality food source than native grass seeds (Kelrick et al., 1986; Lucero et al., 2015; however see: Richardson et al., 2013); thus, individuals in cheatgrass‐dominated habitats may be in worse condition. Foraging costs should be lower for individuals in poorer condition, which may decrease GUD in risky foraging trays (Brown & Kotler, 2004; Kotler et al., 2010) and potentially result in a conservative estimate of predation risk. In addition, risk aversion may vary within a population by age, sex, or reproductive state (Bedoya‐Perez et al., 2013). For the numerically dominant species in our study, deer mice, there were no significant differences between cheatgrass and native habitats in the proportion of juveniles, females, or reproductively active individuals. This minimized the likelihood of a state‐dependent behavior bias due to differences in population structure between sites (Bedoya‐Perez et al., 2013).

Perceived risk and foraging tray encounter rate may be confounded if changes in habitat structure simultaneously influence both processes. For example, the likelihood of a mouse encountering a tray in dense cheatgrass cover may be lower than in less dense native vegetation. We conducted a post‐hoc z‐test for proportions to assess whether the non‐shrub (risky) tray encounter rate differed between cheatgrass and native sites. The proportion of non‐shrub trays visited did not differ significantly (*p *=* *0.34) between cheatgrass and native sites, indicating that our results were likely not driven by differences in encounter rate. Rather, the mean proportion of non‐shrub trays visited was actually slightly higher on cheatgrass sites, yet GUDs were significantly lower. It is also unlikely that mice foraged more under shrubs due to a shrub seed preference since sagebrush seeds are low in nutritional quality and often avoided by granivores such as rodents (Kelrick et al., 1986).

Animals also attempt to mitigate risk via habitat selection (Brown & Kotler, 2004; Lagos et al., 1995). Habitat selection often occurs at nested spatial scales, with selection at a coarse scale constraining what is available for selection at a fine scale (Johnson, 1980). Assessment of habitat selection at a single scale can therefore be misleading (Chalfoun & Martin, 2007; Johnson, 1980). We assessed deer mouse microhabitat selection relative to both micro (powder track scale) and macro (trapping grid or site scale) habitat availability and found consistent results across both scales. In partial agreement with our foraging experiment, deer mice consistently avoided cheatgrass and selected shrub cover at both micro‐ and macro‐scales of habitat availability. In addition, the cheatgrass–shrub interaction at the micro‐scale was not significant but suggested that selection for shrub cover was stronger where cheatgrass cover was greater. These results are consistent with the hypothesis that shrubs decrease (Lagos et al., 1995; Longland & Price, 1991) and cheatgrass increases predation risk for small mammals, potentially by impeding movement (Rieder et al., 2010).

Deer mice also avoided native grass at both spatial scales suggesting that dense cover of grasses in general is not optimal; however, cheatgrass avoidance was considerably stronger than that of native grass (Table 5). It is surprising that deer mice did not select native grasses since rodents, birds, and arthropods have all shown a preference for native seeds over cheatgrass seeds (Goebel & Berry, 1976; Kelrick et al., 1986; Lucero et al., 2015; however, see Richardson et al., 2013). Because we do not know what behavior or activity the powder track represented, it is possible that deer mice avoided areas with high grass cover while moving but preferentially foraged on native grasses.

The cheatgrass–moonlight interaction was only significant at the microhabitat availability scale. The interaction showed avoidance of cheatgrass regardless of moonlight, but avoidance was stronger during the lower than upper quarter which, contrary to the foraging behavior results, implied that cheatgrass decreased perceived risk. This finding does not alter our interpretation, however, because the model had little support and poor predictive ability. In addition, our perceived risk expectation based on moonlight may have been incorrect for certain nights because we did not have cloud cover data.

### 4.2 Linking perceived risk and fitness

Habitat alteration by invasive plants may alter perceived risk but not necessarily fitness (Shields et al., 2014); however, studies rarely quantify both. We found the same interplay between cheatgrass and shrub cover for deer mouse apparent survival as for perceived predation risk. As shrub cover decreased, the negative effect of cheatgrass on apparent survival increased; as cheatgrass cover increased, the positive effect of shrubs on apparent survival increased. These results cumulatively suggest that (1) the increased perceived risk in cheatgrass habitats likely reflected actual risk with negative fitness consequences, and (2) the interactive effect of cheatgrass and shrub cover on apparent survival was likely due to cheatgrass increasing and shrub cover decreasing predation risk although lower food quality in cheatgrass habitats may exacerbate this effect (Kelrick et al., 1986; Lucero et al., 2015). It is unlikely that seed supplementation from the GUD experiment influenced the apparent survival results. The GUD experiment occurred on four high and four low cheatgrass cover sites (Table 2); thus, any seed supplementation effect was distributed across our primary habitat gradient (cheatgrass cover). In addition, the GUD experiment was implemented over a short timeframe (6 days per site) relative to the entire summer trapping season used to estimate apparent survival.

Native plants can moderate the effects of exotic plants on other native plant species (Baohanta et al., 2012); however, to our knowledge, no other study has found evidence that native plants alleviate the effects of invasive plants on wildlife. The previous lack of evidence for such interactive effects may be partly explained by the common use of categorical comparisons (native vs. invasive), which may be unable to detect effects that occur along a continuum (Litt & Pearson, 2013; van Riper, Paxton, O'brien, Shafroth, & Mcgrath, 2008). By providing refuge habitat for small mammals, our results suggest that shrubs may partly offset the likely increase in predation risk due to cheatgrass invasion. One long‐term consequence of cheatgrass invasion, however, is increased fire frequency, which often results in the loss of the shrub community (Balch, Bradley, D'Antonio, & Gómez‐Dans, 2013; D'Antonio & Vitousek, 1992). Shrubs may thus only offset the effects of cheatgrass for small mammals in the short term. Moreover, in shrub‐free sites already converted to cheatgrass monocultures, the negative effect of cheatgrass on particular small mammal species likely increases over time (Ostoja & Schupp, 2009). Time since invasion may therefore add another dimension to cheatgrass, native plant, and animal interactions, which should be assessed in future studies (Ostoja & Schupp, 2009; Strayer, Eviner, Jeschke, & Pace, 2006), as we unfortunately do not know when cheatgrass first invaded our sites.

Finally, our survival results should be interpreted within the context of the model set and the overall results. The second ranked survival model was competitive based on AIC_*c*_, and had a positive quadratic effect of cheatgrass and no effect of shrub cover on deer mouse apparent survival. However, given the agreement between the foraging experiment, habitat selection, and apparent survival results, the *cheatgrass × shrub* apparent survival model is not only the top ranked, but also the most plausible ecologically.

As invasive species transform native habitats, the risk landscape can be altered with unknown fitness consequences for many native species. Quantifying changes in perceived risk by native species is an important first step to understand how habitat changes may alter predator–prey dynamics (e.g., Bishop & Byers, 2015; Dutra et al., 2011; Johnson & De León, 2015). A critical next step is to simultaneously measure fitness outcomes to understand (1) how well perceived risk reflects actual risk, and (2) if changes in the risk landscape are likely to affect fitness and, therefore, population dynamics. Understanding the implications of habitat change due to invasive plants is particularly important given the global scope of plant invasions (van Kleunen et al., 2015) and the wide variety of organisms and ecosystem processes that are affected (Crooks, 2002; Vila et al., 2011). Finally, few studies have shown that native plants can mediate the effects of invasives on wildlife. Once established, the eradication of invasive plants is notoriously difficult (Simberloff et al., 2013). Our results suggest that maintaining key native habitat elements, such as shrub cover, may help offset the effect of invasive plants without necessitating complete removal of the invasive species (van Riper et al., 2008).

## Data Accessibility

Data can be accessed through Dryad (http://datadryad.org) and provisional DOI: 10.5061/dryad.f65h2.

## Conflict of Interest

None declared.
