# Supplementary material for: When perception reflects reality: Non‐native grass invasion alters small mammal risk landscapes and survival
Source: Ecol Evol. 2017 Feb 15;7(6):1823–35. doi: 10.1002/ece3.2785 (PMC5355188; doi:10.1002/ece3.2785)
Supplement: Supplementary file 1 [file ECE3-7-1823-s001.docx]

# Supporting Information

# Appendix S1

*Additional details on study site description, site selection, and adjacent riparian habitat covariate*

We compiled potential sites based on the U.S. Geological Survey ReGap land cover data (USGS 2011), local vegetation data from the Thunder Basin Grasslands Prairie Ecosystem Association (http://tbgpea.org, unpublished report), and recommendations from TBNG biologists. All sites excluded riparian areas, trees and anthropogenic structures such as well pads, although some sites were adjacent to such features. The final sites were selected based on visual assessments of site characteristics along transects, prioritizing a collection of sites that covered a broad range of cheatgrass cover.

Riparian areas can provide small mammals with refuges from predators and abundant food resources; consequently, riparian habitat may act as a population source for adjacent upland habitats (Doyle 1990; Hamilton *et al.* 2015). No site contained riparian habitat, however, multiple sites were adjacent to riparian zones (≥5 m) due to suitable trapping conditions in those areas. To account for a potential riparian effect, we created a 200 m buffer around each site based on home range sizes and dispersal distances of local small mammal species (French, Tagami & Hayden 1968; Douglass *et al.* 2006). Polygons of riparian habitat were digitized at 1:1000 resolution using the National Hydrograph Dataset (USGS 2007, http://nhd.usgs.gov) and NAIP imagery of Wyoming (NRCS-USDA 2012, http://gdg.sc.egov.usda.gov). We calculated the percentage of the buffer that overlapped the riparian polygon for each site (ESRI 2012), which became the riparian covariate value.

# APPENDIX S2

*Additional details on habitat selection field and analysis methods*

Deer mouse habitat selection was assessed in 2013 and 2014 using powder tracking following Stapp (1997) and Lemen & Freeman (1985). On the final morning of a primary period, a subset of primarily adult deer mice (excluding lactating or pregnant females) were removed from the site and placed in a quiet, cool location during the day (n = 35 adults and 3 sub-adults, across 12 of 16 trapping sites). Individuals were kept in a clean trap with fresh bedding and an apple slice for hydration. Approximately 1-2 hours before sunset, mice were coated with fluorescent powder (Radiant Color, Inc.) and released at the location of capture. Powder was applied using a paintbrush to minimize powder inhalation by the mouse (Stapp *et al.* 1994). To target nocturnal rather than initial escape behavior, the track was first followed at approximately sunset in order to mark the last daytime location, which was often a burrow. Starting from the last daytime location, the track was followed beginning 2–4 hours after sunset using an ultraviolet LED flashlight (http://ledwholesalers.com) and flagged every meter.

Used and available microhabitat were surveyed within 1–5 days of tracking for the majority (71%) of deer mice (range: 1–13 days); available macrohabitat (site-level) was surveyed within the same summer as the track.

We tested for sex and reproductive status (scrotal or non-scrotal males) effects by adding each term to the top model after model selection; these predictors were only retained if the model was ranked higher than the same model excluding individual covariates. Goodness-of-fit was assessed using the change in Pearson Χ^2^ (∆Χ^2^) with a cut-off of four (equation 7.8: Hosmer, Lemeshow & Sturdivant 2013), and model performance was measured with concordance.

# Appendix S3

*Additional details on apparent survival field and analysis methods*

Each site had a 135 × 165 m trapping grid consisting of 120 live traps separated by 15 m. The grid configuration of four sites was slightly modified to stay within the primary habitats (trap spacing was unaltered). Each grid had 80 Sherman (H.B. Sherman Traps Inc.) and 40 Havahart traps (Woodstream Corp.) to target a broader suite of species (O’Farrell *et al.* 1994), and each trap was covered with a small plywood board to mitigate the effects of adverse weather. Traps were set in the afternoon and checked the following morning. We baited traps with a rolled oats-peanut butter mixture and provided bedding. Captures were marked with a PIT tag (passive integrated transponder, Biomark Inc.) and sex, age (juvenile, sub-adult, or adult), mass (g), and reproductive status (M: scrotal or not active; F: perforate vagina, pregnant, lactating, or not active) were recorded. Animals were released at the location of capture.

There is no overall goodness of fit test for robust design models. We thus qualitatively assessed goodness of fit with an overdispersion parameter ($\hat{c})$ sensitivity test on the final model set (e.g., McGowan *et al.* 2011; Stauffer *et al.* 2014). We used quasi-likelihood AIC*_c_* (QAIC*_c_*) to assess how model rankings changed with $\hat{c}$ values of 1.5, 2.0 and 3.0*.* Additionally, metrics have not been developed to assess model performance for many mark-recapture models, including robust design; thus, no performance measure was reported.

# Table S1

Complete stage two deer mouse apparent survival (*Φ*) model set from the Huggins robust design analysis. For all models, probability of capture (*p*) = year + age + sex and recapture (*c*) = year + site + age + sex. Models with NA under *w* meet the uninformative predictor criteria. AIC*_c_* = Akaike’s Information Criterion corrected for small sample sizes, *K* = number of parameters, ∆AIC*_c_* = AIC*_ci_* – minimum AIC*_c_*, and *w* = AIC*_c_* model weight

| Model | K | ∆AIC*_c_** | *w_i_* | Deviance |
| --- | --- | --- | --- | --- |
| *Φ*(cheat × shrub + year) | 27 | 0.00 | 0.47 | 6697.22 |
| *Φ*(cheat^2^ + year) | 26 | 1.70 | 0.20 | 6700.97 |
| *Φ*(cheat^2^ + shrub + year) | 27 | 2.11 | 0.16 | 6699.32 |
| *Φ*(cheat + shrub + year) | 26 | 2.23 | 0.15 | 6701.49 |
| *Φ*(cheat + shrub + riparian + year) | 27 | 3.29 | NA | 6700.51 |
| *Φ*(cheat + shrub × year) | 27 | 3.73 | NA | 6700.95 |
| *Φ*(cheat + shrub + cattle + year) | 27 | 4.27 | NA | 6701.49 |
| *Φ*(cheat + effort + year) | 26 | 8.50 | 0.01 | 6707.77 |
| *Φ*(global) | 38 | 10.33 | 0.00 | 6684.93 |
| *Φ*(cheat + low temp + precip) | 26 | 11.16 | 0.00 | 6710.42 |
| *Φ*(cheat + cattle + year) | 26 | 12.11 | 0.00 | 6711.37 |
| *Φ*(cheat + riparian + cattle + year) | 27 | 13.67 | NA | 6710.89 |
| *Φ*(cheat + riparian × year) | 27 | 14.52 | 0.00 | 6711.74 |
| *Φ*(cheat + year) | 25 | 15.08 | 0.00 | 6716.39 |
| *Φ*(cheat + riparian + year) | 26 | 15.74 | 0.00 | 6715.01 |
| *Φ*(cheat × year) | 26 | 16.30 | 0.00 | 6715.56 |
| *Φ*(cheat + natv g + year) | 26 | 17.12 | NA | 6716.38 |
| *Φ*(cheat × riparian + year) | 27 | 17.31 | 0.00 | 6714.53 |
| *Φ*(null) | 23 | 23.37 | 0.00 | 6728.76 |
| *Φ*(year) | 24 | 23.68 | 0.00 | 6727.03 |

*Minimum AIC*_c_* = 6751.85.

# FIGURE S1

Representative photographs of paired foraging trays in a high cheatgrass cover site (A) and a low cheatgrass cover site (B). Within a pair, trays were separated by 1.5 m. Both photographs were taken on June 6, 2014.


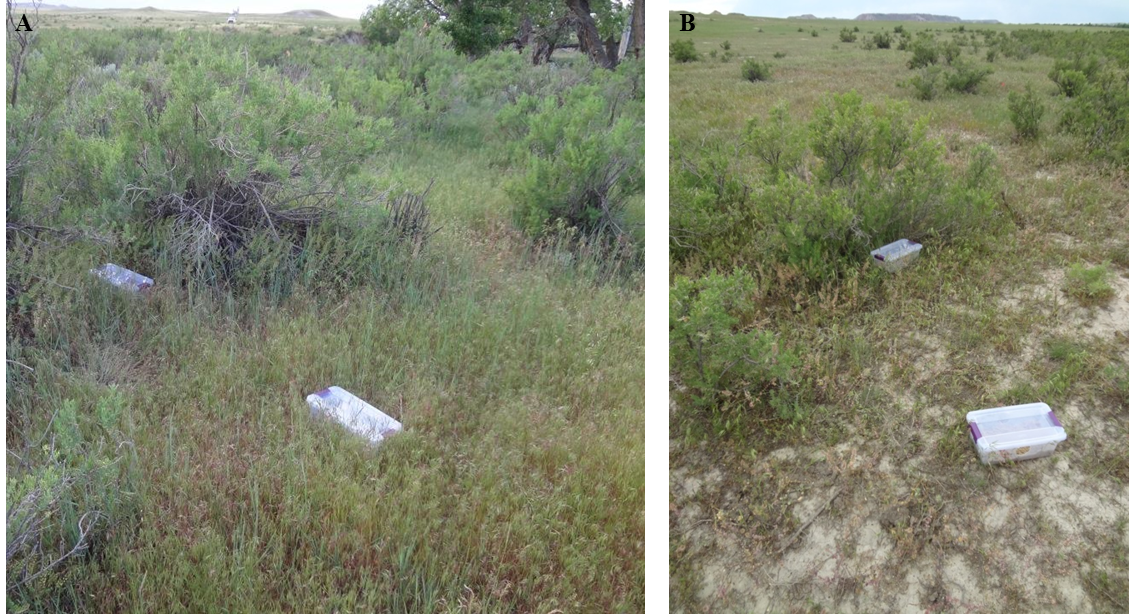


# References

Douglass, R.J., Semmens, W.J., Matlock-Cooley, S.J. & Kuenzi, A.J. (2006) Deer mouse movements in peridomestic and sylvan settings in relation to Sin Nombre virus antibody prevalence. *Journal of wildlife diseases*, **42**, 813–8.

Doyle, A.T. (1990) Use of riparian and upland habitats by small mammals. *Jounal of Mammalogy*, **71**, 14–23.

Environmental Systems Resource Institute (ESRI). (2012) ArcMap 10.1.

French, N.R., Tagami, T.Y. & Hayden, P. (1968) Dispersal in a population of desert rodents. *Jounal of Mammalogy*, **49**, 272–280.

Hamilton, B.T., Roeder, B.L., Hatch, K.A., Eggett, D.L. & Tingey, D. (2015) Why is small mammal diversity higher in riparian areas than in uplands? *Journal of Arid Environments*, **119**, 41–50.

Hosmer, D.W.J., Lemeshow, S. & Sturdivant, R.X. (2013) Logistic regression for matched case-control studies. *Applied Logistic Regression*, 3rd ed, p. John Wiley & Sons, Inc., Hoboken, NJ.

Lemen, C. & Freeman, P. (1985) Tracking mammals with fluorescent pigments: a new technique. *Journal of Mammalogy*, **66**, 134–136.

McGowan, C.P., Hines, J.E., Nichols, J.D., Lyons, J.E., Smith, D.R., Kalasz, K.S., Niles, L.J., Dey, A.D., Clark, N. a., Atkinson, P.W., Minton, C.D.T. & Kendall, W. (2011) Demographic consequences of migratory stopover: linking red knot survival to horseshoe crab spawning abundance. *Ecosphere*, **2**, 1–22.

O’Farrell, M.J., Clark, W.A., Emmerson, F.H., Juarez, S.M., Kay, F.R., O’Farrell, T.M. & Goodlett, T.Y. (1994) Use of a mesh live trap for small mammals: are results from sherman live traps deceptive? *Journal of Mammalogy*, **75**, 692–699.

Stapp, P. (1997) Habitat selection by an insectivorous rodent: patterns and mechanisms across multiple scales. *Journal of Mammalogy*, **78**, 1128–1143.

Stapp, P., Young, J.K., Vandewoude, S. & Horne, B. Van. (1994) An evaluation of the pathological effects of fluorescent powder on deer mice (*Peromyscus maniculatus*). *Journal of Mammalogy*, **75**, 704–709.

Stauffer, G., Rotella, J., Garrot, R. & Kendall, W. (2014) Environmental correlates of temporary emigration for female Weddell seals and consequences for recruitment. *Ecology*, **95**, 2526–2536.

US Geological Survey. (2011) Gap Analysis Program (GAP), National Land Cover, Version 2.
